# The C-terminal domain of Emi2 conjugated to cell-penetrating peptide activates mouse oocyte

**DOI:** 10.3389/fcell.2025.1578020

**Published:** 2025-04-16

**Authors:** Toru Suzuki

**Affiliations:** ^1^ Animal Research Facilities, Institute of Science Tokyo, Tokyo, Japan; ^2^ Laboratory of Genome Editing for Biomedical Research, Medical Research Laboratory, Institute of Science Tokyo, Tokyo, Japan

**Keywords:** oocyte activation, meiotic arrest, cell-penetrating peptide, preimplantation development, mouse

## Abstract

**Introduction:**

The cell cycle of ovulated oocytes from various animal species, including mice, arrests at the second meiotic metaphase until fertilization. The meiotic cell cycle must be initiated to initiate embryonic development. Besides natural fertilization, several methods have been developed to activate unfertilized oocytes without sperm. These methods aid both animal production and molecular studies on meiotic regulation, oocyte activation, and embryogenesis. This study aimed to develop a method to activate mouse oocytes using a cell-penetrating peptide based on the knowledge that the C-terminal domain of the meiotic protein Emi2 can resume the arrested meiotic cell cycle.

**Methods:**

This study used female B6D2F1 mice to investigate the effects of a cell-penetrating peptide-fused Emi2 peptide on oocyte activation. Second meiotic metaphase oocytes were collected, cultured, and treated with the peptide or strontium chloride. Pronuclear formation, second polar body extrusion, and blastocyst development were assessed, and statistical significance was determined using Fisher’s exact test.

**Results:**

The cell-penetrating peptide activated zona-intact oocytes in a manner dependent on specific amino acid residues and peptide concentrations, which are critical components for cell membrane penetration. Some oocytes did not survive after the peptide treatment, indicating its cytotoxic effects. It has also been confirmed that oocytes activated using this method can develop to the blastocyst stage.

**Discussion:**

The introduction of peptides or functional amino acid sequences using cell-penetrating peptide or related methods could be an alternative for easily performing functional analyses of the activity of target proteins in oocytes.

## 1 Introduction

In unfertilized oocytes of various species, including mice and frogs, the cell cycle is arrested at the second meiotic metaphase (MII) until fertilization (Sagata, 1996). Therefore, one of the earliest and most critical events in embryogenesis initiation is the resumption of cell cycle arrest in oocytes. Meiotic arrest and resumption are tightly regulated processes, and any disruption in this regulatory mechanism can severely impair the developmental rate of embryos, potentially leading to developmental arrest or infertility in the most severe cases (Hashimoto et al., 1994; Colledge et al., 1994; Backs et al., 2010; Oh et al., 2011; Nozawa et al., 2023).

The molecular mechanisms that regulate MII arrest and meiotic resumption in unfertilized oocytes have been extensively studied in frogs and mice, and a series of molecular events leading to meiotic resumption have been proposed. The transition from metaphase II to anaphase II in oocytes requires the degradation of cyclin, a maturation promoting factor/M-phase promoting factor (MPF) component, with the assistance of an anaphase-promoting complex (APC) with ubiquitin ligase activity, which is inhibited by Emi2, a zinc finger protein (Tung et al., 2005; Shoji et al., 2006), in unfertilized oocytes. Upon fusion of unfertilized oocytes with sperm, the free calcium ion concentration in the oocyte cytoplasm increases, activating CaMKIIγ, a calcium/calmodulin-dependent kinase (Liu and Maller, 2005). Phosphorylation of Emi2 by activated CaMKIIγ, followed by phosphorylation of the Emi2 degron by Plk1, results in Emi2 degradation and inactivation. The inactivation of Emi2 enhances APC activity, leading to cyclin ubiquitination and degradation and MPF inactivation, ultimately triggering meiotic cell cycle resumption, that is oocyte activation (Liu and Maller, 2005; Madgwick and Jones, 2007).

Besides sperm-mediated oocyte activation, various techniques have been developed for mammalian species to regulate the meiotic cell cycle and artificially stimulate unfertilized oocytes to begin embryogenesis (Nasr-Esfahani et al., 2010). Ethanol (Cuthbertson, 1983), strontium (Whittingham and Siracusa, 1978), and calcium ionophores (Steinhardt and Epel, 1974) are well-known agents used for oocyte activation. Oocyte activation by these agents is associated with molecular events triggered by elevated ooplasmic calcium concentrations that mimic natural fertilization (Fulton and Whittingham, 1978; Fulton and Whittingham, 1978; Cuthbertson et al., 1981). Some oocyte activation methods are employed not only in basic biological research using animal models but also in assisted reproduction technologies to support human reproduction in clinical settings (Tesarik and Sousa, 1995).

Ohe et al. (2010) and Suzuki et al. (2010a) reported that the C-terminal amino acids of Emi2 are essential for inhibiting APC activity in frog and mouse oocytes, respectively. They also demonstrated that the treatment of frog egg extracts with the Emi2 C-terminal peptide (Ohe et al., 2010) or injection of mRNA encoding the Emi2 C-terminal region into unfertilized mouse oocytes (Suzuki et al., 2010a) resumed MII arrest. Based on the observation that the Emi2 C-terminus binds to APC or its component Cdc20 (Ohe et al., 2010; Suzuki et al., 2010a), the Emi2 C-terminal sequence has been proposed to competitively inhibit the binding of endogenous Emi2 to APC in unfertilized oocytes, thereby inducing cell cycle reentry through APC activation and inactivation of M-phase cyclins mediated by endogenous Emi2 inhibition (Ohe et al., 2010; Suzuki et al., 2010a).

Ohe et al. (2010) and Suzuki et al. (2010a) suggested that the Emi2 C-terminal peptide could serve as a novel oocyte activator for mammalian embryo production. However, it remains unclear whether peptides can activate living and intact MII oocytes without inducing undesirable cellular effects that disrupt normal meiotic processes and preimplantation embryo development. To test this hypothesis, it was necessary to introduce the peptide into unfertilized oocytes. Micromanipulation is one of the most effective techniques for introducing molecules into oocytes and embryonic cells (Gordon and Ruddle, 1981; Kimura and Yanagimachi, 1995; Suzuki and Perry, 2018); however, it requires specialized equipment and expertise. Another alternative is the use of cell-penetrating peptides (CPPs). CPPs were first discovered by Frankel and Pabo when studying the HIV viral protein TAT (Frankel and Pabo, 1988). When purified TAT is added to culture media, it is taken up by cultured cells *in vitro*, localized to the nucleus, and activates the viral promoter (Frankel and Pabo, 1988). The CPP sequence of TAT comprises only 13 amino acids (TAT (48–60), Vivès et al., 1997). Since then, several other amino acid sequences of known proteins and artificially designed peptides have been shown to exhibit cell-penetrating activity (Guidotti et al., 2017). Some CPPs contain as few as 13 amino acids and can be chemically synthesized by fusion with target peptides with relative ease.

In this study, the ability of CPP-fused mouse Emi2 C-terminal peptide to induce meiotic resumption in unfertilized mouse oocytes was investigated. These results demonstrated that mouse oocytes with a zona pellucida treated with the CPP-Emi2 C-terminal peptide initiated embryonic development, with some oocytes developing to the blastocyst stage.

## 2 Materials and methods

### 2.1 Animals

All the animal experiments were approved by the Institutional Animal Care and Use Committee of the Institute of Science Tokyo, Japan. Female B6D2F1 mice were obtained from CLEA Japan and maintained under a 12-h light/dark cycle. Gamma-irradiated CE-2 diet (CLEA Japan, Tokyo, Japan) and sterilized water (Hydropac; Natsume Seisakusho, Tokyo, Japan) were provided *ad libitum*.

### 2.2 Design of peptides

The mouse Emi2 protein contains a highly conserved RL tail domain at its C-terminus in several species, including *Xenopus laevis* (Suzuki et al., 2010a; Ohe et al., 2010). It has been reported that a peptide consisting of 14 amino acids from the RL tail of the *Xenopus* Emi2/Erp1 protein can resume the meiotic cell cycle in *Xenopus* egg extract (Ohe et al., 2010). Additionally, the mRNA encoding residues 551–641 of the mouse Emi2 protein activates mouse MII oocytes (Suzuki et al., 2010a). Based on this information, it is suggested that the conserved RL tail of mouse Emi2 can activate mouse MII oocytes, thereby promoting pre-implantation embryo formation.

To test this possibility, a peptide sequence containing 18 residues from the C-terminal end of the mouse Emi2 protein (RL peptide), along with a cell-penetrating peptide (CPP) and 8 N-terminal arginines (R8; Mitchell et al., 2000; Futaki et al., 2001) was designed and synthesized as a putative cell-penetrating oocyte activation peptide (R8-RL peptide; RRRRRRRRSKDVLPGSAQSKRNLKRL). Because most C-terminal arginine and leucine residues of the Emi2 protein are essential for the function of the RL tail (Ohe et al., 2010; Suzuki et al., 2010a), a mutant peptide in which these two residues (RL) were substituted with two alanine residues (AA) was synthesized as a negative control (R8-AA peptide; RRRRRRRRSKDVLPGSAQSKRNLKAA). To investigate the effects of CPP for the function of the RL peptide, RL peptide without R8 was also synthesized (RL peptide; SKDVLPGSAQSKRNLKRL). Peptides were obtained from ABclonal Biotechnology K.K. (Massachusetts, United States) or GL Biochem (Shanghai) Ltd. (Shanghai, China), dissolved in sterilized water (NAKALAI, Kyoto, Japan) at the stock concentration of 30 mM, and stored at −80°C until use.

### 2.3 Oocyte collection and *in vitro* culture

Female B6D2F1 mice, 8–12 weeks of age, were superovulated to collect MII oocytes by sequential injections of 7.5 IU pregnant mare serum gonadotropin (PMSG; Asuka Animal Health, Tokyo, Japan) and 7.5 IU human chorionic gonadotropin (hCG; Asuka Animal Health, Tokyo, Japan) 48 h apart. Then, 15–16 h after hCG injection, the mice were sacrificed by cervical dislocation, and oviducts containing the cumulus-oocyte complexes were collected. Denuded MII oocytes (Supplementary Figure S1A) with a meiotic spindle-associated cortical bump and degenerated first polar body, without nucleus-like structure (Suzuki et al., 2016), were washed and cultured in KSOM medium (ARK Resource, Kumamoto Japan) under 5% CO_2_ at 37°C after cumulus cell removal using 0.29 mg/mL of mouse embryo-tested hyaluronidase (Sigma, Darmstadt, Germany) in M2 medium (Merck, Darmstadt, Germany) for 5 min on heating stage at 37°C. Collected MII oocytes were cultured for a minimum of 1 h prior to the experiments to confirm the absence of spontaneous activation associated with the extrusion of the second polar body, as observed under microscope IX73 (Olympus, Tokyo, Japan) or SMZ-1500 (Nikon, Tokyo, Japan). Polar bodies extruded from activated MII oocytes with degenerated first polar body were recognized as second polar bodies (Suzuki et al., 2016).

### 2.4 Microinjection of peptides

Glass needles were fabricated using thin wall borosilicate glasses (B110-75–10; Sutter Instrument Co., CA), a micro pipette puller (P-97; Sutter Instrument Co., CA) and a micro forge (MF2; Narishige, Tokyo, Japan). For the injection experiments, about 10 pL of R8-RL or R8-AA peptides dissolved in sterilized water (NAKALAI, Kyoto, Japan) at the indicated concentrations (30 or 3 mM) were injected into MII oocytes in an M2 medium using a micro glass needle, approximately 1 µm in internal diameter, connected with piezo-actuated micromanipulator (PMM-150, Primetech, Ibaraki, Japan). The Piezo drive control was set to an intensity of 2-3 and a speed of 1. The extrusion of the second polar body, pronuclear formation, dead oocytes, and MII oocytes were observed under microscopes 6–7 h after injection.

### 2.5 Oocyte treatment with peptides with or without zona pellucida incision

Physical incision (30–50 µm) of the zona pellucida of MII oocytes was performed in an M2 medium using a glass needle (B110-75-10; Sutter Instrument Co., CA) with a sharpened tip attached to a micromanipulator (Narishige, Tokyo, Japan). For peptide treatment, zona-intact and incised MII oocytes were cultured in KSOM containing the indicated concentrations of peptides (3–0.3 mM) for 2.5 h. The second polar body extrusion, pronuclear formation, dead oocytes and MII oocytes were observed and counted under microscopes 6–7 h after the initiation of the peptide treatment. Blastocyst development with a cavity was assessed 5 days after the initiation of oocyte activation with peptides. Untreated oocytes were cultured as control.

### 2.6 Oocyte treatment with peptides or strontium in the presence of cytochalasin B

To test the preimplantation developmental potential of diploid embryos, zona-intact MII oocytes were treated with peptides in the presence of a cytokinesis inhibitor, cytochalasin B, at the concentration of 5 μg/mL (FUJIFILM Wako, Tokyo, Japan), for 2.5 h. As a control of conventional oocyte activation method, MII oocytes were treated with 10 mM strontium chloride (Merck, Darmstadt, Germany) in a calcium-free and 1 mg/mL glucose-supplemented CZB medium (Chatot et al., 1989) containing 5 μg/mL of cytochalasin B for 2.5 h to induce diploid parthenogenotes. After the treatment with a cytochalasin B-containing medium, peptide- or strontium-treated MII oocytes were washed and further cultured in KSOM with 5 μg/mL cytochalasin B for 4.5 h. The resulting embryos were washed and cultured in KSOM for 5 days. Pronuclear formation and second polar body extrusion were observed at 6–7 h after the initiation of oocyte activation treatment under microscopes. Blastocyst development with a cavity was assessed 5 days after the initiation of oocyte activation with peptides or strontium. Untreated oocytes were cultured as control.

### 2.7 Statistics

Fisher’s exact test was performed, and p-values less than 0.05 were considered significantly different. For comparisons involving more than three groups, p-values were adjusted using the Holm correction. All oocyte activation experiments were conducted in at least two independent replicates.

## 3 Results

### 3.1 Design and activity of Emi2 C-terminal peptide

First, R8-RL or R8-AA peptides, at different concentrations, were directly injected into MII oocytes by micromanipulation to analyze their effects on meiotic arrest (Supplementary Table S1). At a concentration of 30 mM, the R8-RL peptide activated 91.7% ± 1.7% of surviving oocytes (n = 25), as evidenced by pronuclear formation, which was significantly different (p = 2.3 × 10^−11^) from the R8-AA peptide (5.6% ± 5.6%, n = 35). At lower peptide concentrations, the R8-RL peptide did not show significant differences in oocyte activation compared to the R8-AA control (p = 1.0 for 3 and 0.3 mM).

### 3.2 Zona intact oocytes are activated with RL peptide

To investigate whether the CPP-peptides designed in this study affect the meiotic cell cycle of MII oocytes encased by the zona pellucida, MII oocytes with intact or removed zona pellucida were cultured in a medium containing 3 or 0.3 mM RL peptide for 6 h (Table 1). The R8-RL peptide at the concentration of 3 mM activated not only the surviving MII oocytes with severed zona pellucida with a micro glass needle (100%, n = 9) but also those with intact zona pellucida (100%, n = 7) with a significant difference from the R8-AA peptide (p = 8.0 × 10^−8^ for zona-intact oocytes and 8.4 × 10^−6^ for zona-cut oocytes, RL vs. AA). Some MII oocytes died after treatment with these peptides in both the intact and zona-cut groups, indicating their cytotoxicity. At a peptide concentration of 0.3 mM, no significant differences were detected both in zona-intact and cut groups (p = 0.0515 for zona-intact and 0.3833 for zona-cut).

**TABLE 1:** Oocyte activation after the treatment with cell-penetrating peptides, with or without zona pellucida.

Peptide concentration (mM)	3				0.3			
Zona	Intact		Cut		Intact		Cut	
Emi2 C-terminal	RL	AA	RL	AA	RL	AA	RL	AA
Replicates	2	2	2	2	2	2	2	2
Total number of oocytes	37	37	35	36	37	37	35	37
Number of dead oocytes (% ± s.e.m.)*^,^ ^a^	28 (76.2 ± 6.2)	9 (24.3 ± 0.7)	28 (79.9 ± 3.4)	10 (26.9 ± 8.1)	14 (36.8 ± 13.2)	0 (0.0)	12 (34.2 ± 4.7)	0 (0.0)
Number of surviving oocytes	9	28	7	26	23	37	23	37
Number of activated oocytes (%±s.e.m.)*^,^ ^b^	9* (100 ± 0.0)	1 (3.3 ± 3.3)	7* (100 ± 0.0)	2 (7.7 ± 0.0)	3 (15.0 ± 15.0)	0 (0.0)	1 (4.2 ± 4.2)	0 (0.0)
Number of MII oocytes	0	27	0	24	20	37	22	37

*p < 0.05, RL vs. AA in same peptide concentration and zona condition.

Fisher’s exact test was performed on the activated oocytes within the surviving oocytes. RL, R8-RL, peptide; AA, R8-AA, peptide; MII, the second meiotic metaphase.

^a^ % (±s.e.m.) of total number of oocytes.

^b^ % (±s.e.m.) of surviving oocytes.

### 3.3 *In vitro* development of MII oocytes activated with RL peptide

The *in vitro* developmental capacity of oocytes activated by R8-RL peptide was examined. All MII oocytes treated with the R8-RL peptide at any concentration tested (3–0.5 mM) survived after 1 h of treatment, but up to 29.3% ± 0.7% (n = 50–105) were dead after 2.5 h (Supplementary Figure S1B; Supplementary Table S2). All of the concentrations from 3 to 0.5 mM tested in this study activated a part of surviving MII oocytes (39.4% ± 6.1% to 84.8% ± 15.2% of surviving oocytes, n = 34 to 68, Supplementary Figures S1C–E). Of the activated oocytes, 15.4% ± 6.3% to 43.0% ± 2.6% had a second polar body (Supplementary Figure S1D), and 18.8% ± 18.8% to 56.7% ± 10.0% were developed to the blastocyst stage after 5 days of *in vitro* culture (Table 2; Supplementary Figures S2A, C, D, F). For blastocyst development, no significant difference was detected statistically between peptide-treated groups.

**TABLE 2:** Oocyte activation and *in vitro* development after the peptide treatment.

Peptide concentration (mM)	3	2	1	0.75	0.5	0
Replicates	2	2	4	2	2	4
Total number of oocytes	53	49	98	48	50	57
Number of dead oocytes (% ± s.e.m.)*^,^ ^a^	19 (36.6 ± 2.3)	10 (19.6 ± 4.6)	30 (30.9 ± 3.4)	8 (23.4 ± 7.4)	4 (8.0 ± 4.0)	0 (0.0)
Number of surviving oocytes	34	39	68	40	46	57
Number of pronuclear formation (% ± s.e.m.)*^,^ ^b^	27 (84.8 ± 15.2)	27 (69.4 ± 1.2)	34 (51.3 ± 5.5)	26 (64.9 ± 1.8)	18 (39.4 ± 6.1)	0 (0.0)
Number of pronuclear formation with 2nd polar body (% ± s.e.m.)*^,^ ^b^	8 (15.4 ± 6.3)	13 (27.0 ± 9.4)	29 (43.0 ± 2.6)	17 (37.5 ± 0.6)	15 (28.6 ± 7.8)	0 (0.0)
Number of MII oocytes	7	12	34	14	28	57
Number of Blastocyst (% ± s.e.m.)*^,^ ^c^	6 (18.8 ± 18.8)	15 (56.7 ± 10.0)	11 (32.9 ± 6.2)	11 (43.5 ± 14.9)	9 (50.0 ± 0.0)	0 (0.0)

For preimplantation developmental capacity, the number of embryos that developed to the blastocyst stage and those that did not, among the oocytes that formed pronuclei, were analyzed using the Fisher’s exact test, followed by Holm correction to adjust the p-values. The significant difference (p < 0.05) was not detected. MII, the second meiotic metaphase.

^a^ % (±s.e.m.) of total number of oocytes.

^b^ % (±s.e.m.) of surviving oocytes.

^c^ % (±s.e.m.) of pronuclear embryos.

To analyze the developmental potential of diploid embryos, MII oocytes were treated with R8-RL peptide in the presence of an actin polymerization inhibitor, cytochalasin B (CB), to block polar body extrusion and produce diploid embryos. It was initially confirmed (Supplementary Table S3) that 2 and 3 mM of RL peptides without R8 residues, in the presence of CB, did not activate surviving zona-intact MII oocytes (0% activation, n = 50 for each concentration), whereas peptides containing the R8 residues did activate oocytes (p = 1.6 × 10^−14^ for 3 mM and p = 1.8 × 10^−5^ for 2 mM). After treatment with 2 to 0.5 mM R8-RL peptide, 49.1% ± 29.1% to 76.5% ± 8.1% of surviving oocytes (n = 27–48) formed pronuclei but did not exhibit a second polar body (Supplementary Figure S1E), suggesting the production of diploid parthenotes (Table 3). Of the activated oocytes, 6.3% ± 6.3% to 91.7% ± 8.3% developed to the blastocyst stage (Supplementary Figures S2B, E). All of the surviving oocytes treated with both strontium and cytochalasin B (n = 46) were activated and 100% of them developed to the blastocyst stage. The developmental ability of strontium-activated diploid embryos to reach the blastocyst stage was significantly higher than that of all groups activated with the R8-RL peptide (p < 0.05). Embryos activated with the peptide at a concentration of 0.5 mM exhibited superior developmental potential compared to those activated with 1 mM and 2 mM concentrations (p ≦ 0.006, Table 3).

**TABLE 3:** Oocyte activation and *in vitro* development after the peptide treatment in the presence of CB.

Peptide concentration (mM) or Sr	Sr	2	1	0.5	0
Replicates	2	2	2	2	2
Total number of oocytes	50	50	50	50	30
Number of dead oocytes (% ± s.e.m.)*^,^ ^a^	4 (8.0 ± 4.0)	23 (46.0 ± 6.0)	18 (36.0 ± 12.0)	2 (4.0 ± 4.0)	0 (0.0)
Number of surviving oocytes	46	27	32	48	30
Number of MII oocytes	0	13	8	25	30
Number of pronuclear formation (% ± s.e.m.)*^,^ ^b^	46 (100.0 ± 0.0)	14 (51.7 ± 1.7)	24 (76.5 ± 8.1)	23 (49.1 ± 29.1)	0 (0.0)
Number of Blastocyst (% ± s.e.m.)*^,^ ^c^	46^abc^ (100.0 ± 0.0)	1^ad^ (6.3 ± 6.3)	10^be^ (40.6 ± 13.3)	20^cde^ (91.7 ± 8.3)	0 (0.0)

For preimplantation developmental capacity, the number of embryos that developed to the blastocyst stage and those that did not, among the oocytes that formed pronuclei, were analyzed using Fisher’s exact test, followed by Holm correction to adjust the p-values. Significant differences were observed between groups denoted by the same superscript: a (p = 9.2 × 10^−13^), b (p = 3.5 × 10^−8^), c (p = 0.0338), d (p = 8.0 × 10^−6^) and e (p = 0.006). CB, Cytochalasin B; Sr, Strontium; MII, the second meiotic metaphase.

^a^ % (±s.e.m.) of Total.

^b^ % (±s.e.m.) of surviving oocytes.

^c^ % (±s.e.m.) of pronuclear embryos.

## 4 Discussion

The results of this study demonstrated that the CPP (R8)-conjugated mouse Emi2 RL domain peptide, composed of 26 amino acid residues, can activate zona-intact mouse MII oocytes while preserving their preimplantation developmental potential. Peptide-mediated oocyte activation using the peptide designed in this study occurs in a CPP-, RL residue-, and concentration-dependent manner, resulting in the formation of blastocyst-stage embryos after *in vitro* culture.

CPPs have been identified in several cell-penetrating proteins and have been investigated to introduce various molecules that control cellular functions in living cells (Heitz et al., 2009). This technique has already been applied in studies using unfertilized mammalian oocytes and preimplantation embryos (Kwon et al., 2009; Kwon et al., 2013; Klinsky et al., 2023), but reports vary as to whether sufficient transduction efficiency can be achieved. It has been reported that CPP-conjugated LDP12–EGFP–LC3 is not incorporated into mouse MII oocytes enough to detect fluorescent signals in ooplasm (Kwon et al., 2013). Another recent report showed that a CPP-conjugated protein (a permeable tetanus toxin light chain, CPP-TeTx) can move into mouse oocytes regardless of the presence of the zona pellucida, leading to the expected inhibition of cortical granule exocytosis during fertilization (Klinsky et al., 2023). CPP has been reported to cross the cell membrane into the cytoplasm (Frankel and Pabo, 1988; Vivès et al., 1997); however, at the outset of this study, it was unknown whether CPP-conjugated peptides or proteins could cross the zona pellucida, an extracellular matrix, to access the oocytes. The results of this study support the conclusion drawn by Klinsky et al. (2023) that CPP-conjugated proteins can access zona-intact oocytes. The number of amino acids constituting the cargo protein introduced into the oocytes, the concentration of CPP proteins, and the CPP sequences may have a significant effect on the efficiency of intracellular transduction by CPP. Considering that concentrations of 0.5 mM or higher were required for the Emi2 RL peptide (18 amino acids) to activate unfertilized mouse oocytes, and, likely, the introduction of a sufficient number of molecules by CPP in the cell depends on the nature of the cargo being introduced. Some proteins or peptides conjugated to CPP, such as TeTx, exhibit sufficient activity at lower concentrations (4 µM) to regulate cellular functions (Klinsky et al., 2023). However, other compounds, such as 100 mg/mL LDP12–EGFP, display reduced activity to enter mouse oocytes or the two-cell stage embryos (Kwon et al., 2013), necessitating higher concentrations to effectively transfer CPP-proteins or peptides and regulate target molecules, which can result in increased cytotoxicity in this study. It is important to find a way to control the cytotoxicity of CPP-cargo conjugates or increase their activity in cells (Reissmann, 2014; Kim et al., 2021), which is an obstacle to overcome for their widespread use in cell and/or fertilization research. Since it was shown in this study that peptide concentration and treatment time are critical parameters for higher survival and development rates of treated oocytes, their adjustment is necessary to establish better experimental conditions. Some peptides, including Emi2 RL, would require higher concentrations to act on oocytes to regulate cellular processes. Additionally, it may be beneficial to use other CPP sequences with lower toxicity, substances that promote the intracellular delivery of peptides, and reagents that facilitate the repair of damaged cell membranes during CPP penetration.

This study indicates that the Emi2 RL domain peptide triggers sufficient activity to activate MII oocytes and produce blastocyst-stage embryos. Previous reports (Suzuki et al., 2010a; Ohe et al., 2010) suggest that the Emi2 RL peptide introduced into unfertilized mouse oocytes functions as a negative endogenous Emi2 regulator, which is sufficient to produce embryos. It has previously been shown that a zinc ion chelator, TPEN, can also activate mouse MII oocytes (Suzuki et al., 2010b; Kim et al., 2010). The Emi2 protein has a zinc finger domain that is thought to be essential for Emi2 as an APC/C inactivator and has been proposed to remove zinc from MII oocytes, leading to meiotic resumption through Emi2 inactivation and APC/C activation. The proposed mechanism of meiotic resumption is similar to treatment with the Emi2 RL peptide and TPEN in terms of Emi2 function inactivation. The developmental potential from the 1-cell to blastocyst stage of parthenogenetic diploid embryos produced by the Emi2 RL peptide in this study was comparable to that produced by TPEN in a previous study (Suzuki et al., 2010b). The direct functional regulation of target factors downstream of calcium signaling by RL peptides or TPEN allows for a more detailed analysis of the function of meiotic cell cycle regulatory molecules and fertilization-associated events. For example, the finding that TPEN can induce oocyte activation reveals the essential role of zinc in MII arrest. Furthermore, it has been suggested that the only developmentally essential role of calcium oscillation during fertilization is to induce meiotic resumption, since TPEN-mediated oocyte activation is not associated with an ooplasmic calcium pulse but can be used to produce fertile mice. The activity of the Emi2 RL peptide demonstrated in this study supports the idea that the endogenous mouse Emi2 RL domain, which comprises fewer than 18 amino acids, is involved in the maintenance of MII arrest.

The use of chemical inhibitors is a powerful way to control the function of molecules in cells and reveal their intracellular functions. However, there are many proteins, including Emi2, for which no inhibitors have been reported and the low substrate specificity of these chemical inhibitors can be problematic. Molecular control technologies based on the principle of protein-protein interactions, such as the peptides used in this study, can be relatively easily designed from naturally occurring amino acid sequences based on an understanding of the mechanisms of protein behavior and are expected to have higher substrate and interaction specificity (Zutshi et al., 1998; Lu et al., 2020). Antibodies (Biocca and Cattaneo, 1995) and dominant-negative mutant proteins (Benezra et al., 1990) have also been used to regulate the function of target cellular proteins. However, the production of these proteins and their introduction into cells incurs high costs. In cases where CPPs are not used, direct microinjection of proteins or mRNA, genetic expression of these proteins, or other methods are employed or investigated to introduce them into oocytes or embryonic cells. In cells such as unfertilized oocytes or embryonic cells at the earliest developmental stages, where gene expression has not yet occurred (Moore et al., 1974; Bouniol-Baly et al., 1999; Minami et al., 2007), and some essential proteins have already accumulated in sufficient quantities (Minami et al., 2007) to support the earliest developmental events, genetic depletion, and RNA degradation-inducing techniques often exhibit reduced or no efficiency when attempting to inhibit the function of targeted proteins. The introduction of peptides or functional amino acid sequences using CPP or related methods could be an alternative for easily performing functional analyses of the activity of target proteins in oocytes. However, as shown in this study, the CPP-peptide could induce oocyte death. Additionally, in the absence of cytochalasin B, the oocytes activated by the CPP-peptide in this study (Table 2) were assumed to be a mixture of haploid and diploid parthenogenotes. This assumption was based on the observation that these oocytes contained pronuclei, with or without a second polar body, suggesting aberrant regulation of meiotic processes, such as abnormal extrusion of the second polar body. It is likely that meiotic abnormalities, such as aberrant polar body extrusion during oocyte activation, could lead to the formation of embryos with complex developmental outcomes during preimplantation stages (Table 2), supported by reports indicating low developmental potential of embryos with haploid genome and other abnormal karyotypes (Kaufman and Sachs, 1975; Henery and Kaufman, 1992). In the experiment where diploid embryos were produced using the CPP-peptide and CB, the developmental potential to the blastocyst stage varied significantly between activation methods, and different peptide concentrations (Table 3), suggesting that the high concentration of peptide may have toxic effects on embryonic development following oocyte activation. Mitigating or eliminating these detrimental effects of CPPs on living oocytes remains a significant challenge.

## Data Availability

The original contributions presented in the study are included in the article/Supplementary Material, further inquiries can be directed to the corresponding author.

## Supplementary material

SUPPLEMENTARY FIGURE S1 Oocyte activation with an intact zona pellucida using a CPP-conjugatedEmi2 RL domain peptide. **(A)** Representative image of an MII oocyte with a degenerated first polar body before activation. The black scale bar represents 50 μm for panels **(A–E)**. **(B–E)** Representative images of oocytes 6 hours after the initiation of activation using a CPP-conjugated Emi2 RL domain peptide (2 mM). **(B)** Degenerated oocyte. **(C)** Non-activated MII oocyte. **(D)** One-cell parthenogenetic embryo with a single pronucleus and a second polar body. **(E)** One-cell parthenogenetic embryo with two pronuclei and no second polar body. Black arrowheads indicate the degenerated first polar body. White arrowheads indicate the characteristic cortical bump of MII oocytes. The black arrow indicates the second polar body, while the white arrow indicates pronuclei with nucleolus-like structures.

SUPPLEMENTARY FIGURE S2 Blastocyst development following oocyte activation with an intact zonapellucida using a CPP-conjugated Emi2 RL domain peptide. **(A, B)** Representative images of blastocysts observed 5 days after the initiation of oocyte activation using the peptide (2 mM), either in the absence **(A)** or presence **(B)** of cytochalasin B. Both haploid (1-cell embryos with second polar body) and diploid (1-cell embryos without second polar body) embryos were cultured in **(A)**. **(C)** Untreated MII oocytes cultured for 5 days after collection. **(D–F)** Magnified images of panels **(A, B, C, D, E, F)**. The black scale bar in panel **(F)** represents 50 μm for panels **(D, E, F)**.
