# Supplementary material for: The C-terminal domain of Emi2 conjugated to cell-penetrating peptide activates mouse oocyte
Source: Front Cell Dev Biol. 2025 Apr 16;13:1578020. doi: 10.3389/fcell.2025.1578020 (PMC12040968; doi:10.3389/fcell.2025.1578020)
Supplement: Supplementary file 1 [file Table1.docx]

Supplementary Material

# Supplementary Tables

Supplementary Table S1. Oocyte activation after peptide injection into mouse unfertilized oocytes.

| Peptide concentration (mM) | 30 | 3 | 0.3 |
| --- | --- | --- | --- |
| AA  (%±s.e.m.) | 2/35  (5.6±5.6) | 1/30  (4.5±4.5) | 2/37  (5.4±0.1) |
| RL  (%±s.e.m.) | 23/25*  (91.7±1.7) | 2/31  (6.6±1.1) | 2/38  (5.3±0.3) |

* p=2.3 × 10⁻¹¹, RL vs AA. Experiments were performed in 2 independent replicates.

Supplementary Table S2. Effect of treatment duration for oocyte survival.

| Peptide concentration (mM) | 3 | 2 | 1 | 0.75 | 0.5 | 0 |
| --- | --- | --- | --- | --- | --- | --- |
| Replicates | 2 | 2 | 4 | 2 | 2 | 4 |
| Total number of oocytes | 55 | 55 | 105 | 50 | 50 | 57 |
| Number of dead oocytes at 1 h | 0 | 0 | 0 | 0 | 0 | 0 |
| Number of dead oocytes at 2.5 h  (%±s.e.m.) | 16  (29.3±0.7) | 12  (20.4±5.4) | 33  (27.8±3.3) | 10  (20.0±4.0) | 2  (4.0±0.0) | 0  (0.0) |

Supplementary Table S3. Effect of CPP conjugation on oocyte activation.

| Peptide concentration (mM) | 3 | | 2 | |
| --- | --- | --- | --- | --- |
| CPP conjugation | + | - | + | - |
| Replicates | 2 | 2 | 2 | 2 |
| Total number of oocytes | 50 | 50 | 50 | 50 |
| Number of dead oocytes  (%±s.e.m.)^*1^ | 41  (82.0±6.0) | 0  (0.0) | 19  (38.0±2.0) | 0  (0.0) |
| Number of surviving oocytes | 9 | 50 | 31 | 50 |
| Number of MII oocytes | 0 | 50 | 21 | 50 |
| Number of pronuclear formation  (%±s.e.m.)^*2^ | 9^a^  (100.0±0.0) | 0  (0.0) | 10^b^  (31.9±11.9) | 0  (0.0) |

Fisher's exact test was performed on the activated oocytes within the surviving oocytes. Significant differences were observed between CPP+ and -in same peptide concentration, denoted by letters: a (p=1.6x10^-14^) and b (p=1.8x10^-5^). CPP, cell-penetrating peptide (8 arginine); MII, the second meiotic metaphase.

^*1^% (±s.e.m.) of total number of oocytes.

^*2^% (±s.e.m.) of surviving oocytes.
