# Supplementary figures and images for: The C-terminal domain of Emi2 conjugated to cell-penetrating peptide activates mouse oocyte
Source: Front Cell Dev Biol. 2025 Apr 16;13:1578020. doi: 10.3389/fcell.2025.1578020 (PMC12040968; doi:10.3389/fcell.2025.1578020)

Supplemental figure 1

A

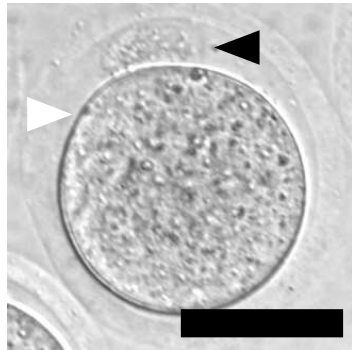

B

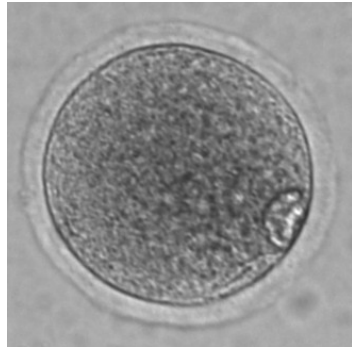

C

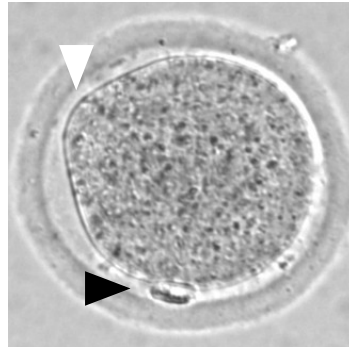

D

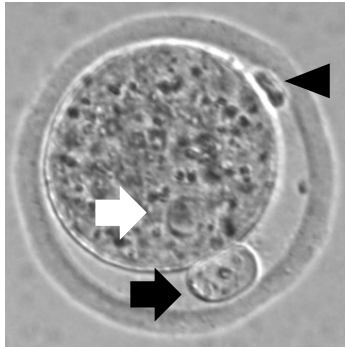

E

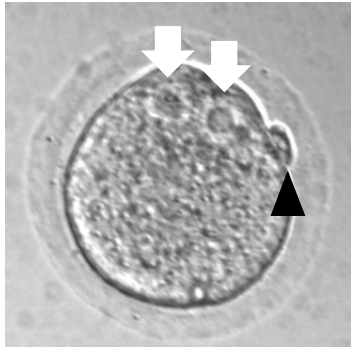

Supplemental figure 2

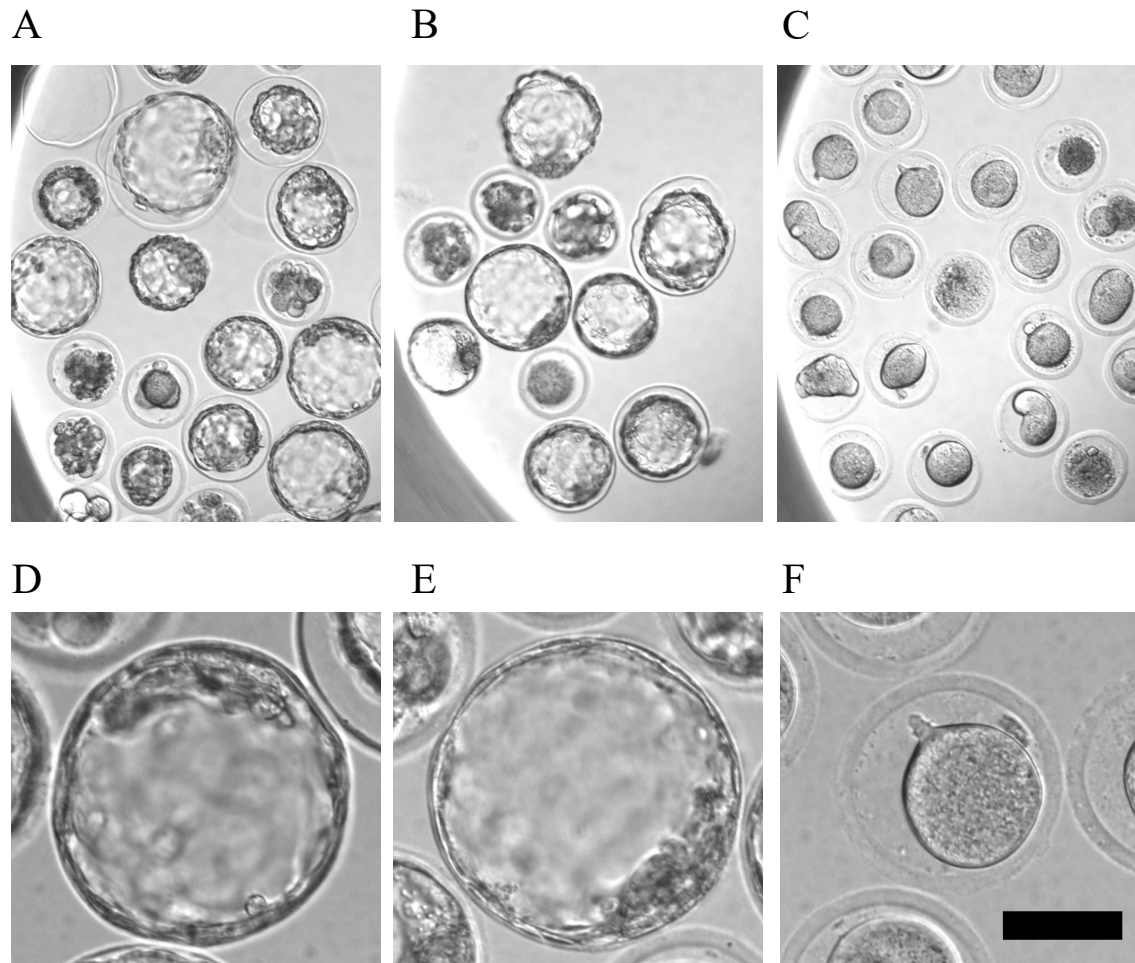

Supplement: Supplementary file 2 [file DataSheet1.pdf]
